# Revealing of Core Shell Effect on Frequency-Dependent Properties of Bi-based Relaxor/Ferroelectric Ceramic Composites

**DOI:** 10.1038/s41598-018-32133-7

**Published:** 2018-09-20

**Authors:** Mohsin Saleem, Lim Dong Hwan, In-sung Kim, Min-Soo Kim, Adnan Maqbool, Umair Nisar, Syed Atif Pervez, Umer Farooq, Muhammad Umer Farooq, Hafiz Muhammad Waseem Khalil, Soon-Jong Jeong

**Affiliations:** 1 Battery Research Center, Korea Electro-technology Research Institute (KERI), Changwon, 641-120 Republic of Korea; 2 School of Chemical and Material Engineering, National University of Science and Technology, Islamabad, Pakistan; 3 Department of Metallurgical & Materials Engineering, University of Engineering and Technology, Lahore, Pakistan; 4 Center of Advanced Materials (CAM), Qatar University, Doha, Qatar; 5 Department of chemical and petroleum engineering, university of Calgary, Alberta, Canada; 6 Department of mechanical engineering, Khwaja Fareed university of engineering and information technology, Rahim yar khan, Punjab Pakistan; 7 Department of Electrical Engineering, UCET, University of Sargodha, Sargodha, Pakistan

## Abstract

In this study, electromechanical characteristics of (1-x) Bi_0.5_Na_0.5_TiO_3_–xSrTiO_3_ (ST26, x = 0.26)/(1-y) Bi_0.5_Na_0.5_TiO_3_–ySrTiO_3_ (ST10, y = 0.1) (matrix/seed) composites were studied. The ST26 (high relaxor phase) and ST10 (a relaxor ferroelectric (RF), high ferroelectric phase) composite with large (r-ST26-ST10) and small (t-ST26-ST10) grains exhibited frequency-related dielectric properties and large strain response at a low triggering electric field (an incipient piezoelectricity). It is ascribed to a matrix-seed effect originating from the inhomogeneous composition due to the presence of two phases. The r-ST26-ST10 composite sintered at 4 h, prominent material, showed a high normalized dynamic strain (d_33_*) of ~700 pm/V (large grains) with stable frequency dependence properties at a low field of 40 kV/cm. The properties of the r-ST26-ST10 composite exhibit less decay with frequency-related polarization and strain compared to those of t-ST26-ST10 composite. The increase in soaking time promotes the diffusion and homogenization of the microstructure in composites, leading to changes in the core-shell structure in the solid solution. The polarization and strain of the ST26-ST10 composites with the frequency are linked to the stability of the internal random fields created by non-ergodic relaxor phase of seed and the amount of phase change in the ergodic relaxor matrix.

## Introduction

Multifunctional ceramics are widely popular in electrical technology devices because of their high electrical, mechanical, optical, and magnetic properties. The piezoelectric ceramics have the capacity to change mechanical energy to electrical energy or vice versa in numerous multifunctional electrical devices, such as actuators and sensors. However, due to environmental concerns, international rules and restriction of certain hazardous substances (RoHS) directives have been applied to decrease the usage of dangerous and toxic substances, especially lead-based materials, which has been recognized as a harmful substance for human health and the environment. Currently, many efforts are underway to enhance the electromechanical performance of lead-free ceramics. However, the lead zirconium titanate (PZT) performance is still better than that of lead-free ceramics^1–4^.

The most effective alternative candidates to PZT are lead-free Bi_0.5_Na_0.5_TiO_3_ (BNT) piezoelectrics, due to their excellent piezoelectric and ferroelectric characteristics and environmental compatibility^4–6^. Lead-free Bi-based systems exhibit quite large strains close to morphotropic phase boundary (MPB) region. The MPB is considered crucial in tailoring the properties, where two phase structures (e.g. tetragonal and rhombohedral) are combined to achieve enhanced ferroelectric properties^4–9^. In particular, lead-free material leads to a phase transition from the ergodic relaxor (ER) phase to long-range ferroelectric (FE) phase with an increasing applied voltage ultimately exhibiting high strain behavior and is a very promising candidate in the actuator industry^7–12^. However, they have the drawback of a large E_c_ (coercive voltage)^8,13–15^. For this purpose, BNT-based system is often modified with other perovskite compounds such as Bi_0.5_K_0.5_TiO_3_ (BKT)^8^, SrTiO_3_ (ST)^7,9–11^, BaTiO_3_ (BT)^7–11^, SrZrO_3_ (SZ)^15^, BiAlO_3_ (BA)^12,13^, and (K,Na)NbO_3_ (KNN)^1–4,14^.

Recently, Acosta *et al*.^7^ synthesized the Bi_0.5_Na_0.5_TiO_3_-SrTiO_3_ (BNT–ST) ceramic system and reported a large normalized dynamic strain (d_33_* ~ 600 pm/V) at a low triggering voltage. MPB region in BNT–ST system was shown to reduce the electric field required to trigger the phase transition at a low voltage for a high strain^7–9^. To utilize the ST in the BNT, the compositionally induced ferroelectric to relaxor phase transition temperature (T_F-R_) is adjusted below room temperature, leading to a low electric field required to achieve a high strain^12^. Further, Sakata and Masuda^10^ studied the BNT–ST system phase change with the increment of the ST amount and investigated the temperature dependent phase transition behavior under applied voltage. Owing to the thermal stability of the ferroelectric BNT-ST based ceramics, few promising systems exhibited properties that are less sensitive to temperature, a high induced strain at low voltage was observed in the entire measured range of temperatures rather than at specific temperatures, which may correspond to the existence of ergodic relaxor (ER) and non-ergodic relaxor (NR) phase regions^16,17^.

Nevertheless, these phases are highly dependent on the frequency-related polarization and strain which limit their usage in real applications^17–19^. To overcome these issues and to enhance the properties, composite of Bi-based solid solutions can be utilized. In composites, there was a decrease in the triggering voltage to enhance the optimum electromechanical properties^13,20–22^. Therefore, the use of composites is very important for electromechanical properties due to the existence of two types of phases. Composites are a mixture of relaxor and ferroelectric phases with the essential polarization and strain factors. The microstructure and crystal configuration of the composite can be tailored by altering the heat treatment^12,22^. Different types of grains in the composite are expected to be evolved at different sintering times to create two types of microstructures and which affect significantly in the polarization and strain behavior. This criterion makes it feasible for the concentration of the matrix to induce the phase transition, resulting in a large strain. Increasing the sintering time in the case of composites produces large or larger grains, and the diffusion of some elements varies in the composition of the two phases between the seed and matrix. Thus, how two-phase composition of composite influences the properties with increasing the sintering time, should be identified. To determine the correlation of the composition and microstructure, raw powders of different grain sizes from different heat treatments (sintering time in this report) were chosen to enhance the electromechanical properties. The frequency dependence of the piezoelectric and ferroelectric properties of the composites at different sintering times were also elaborated in this study to fully understand the role of the seeds in the matrix.

In this report, (1-x) Bi_0.5_Na_0.5_TiO_3_–xSrTiO_3_ (ST26, x = 0.26) composites with seeds of (1-y) Bi_0.5_Na_0.5_TiO_3_–ySrTiO_3_ (ST10, y = 0.1) were synthesized by a conventional solid-state reaction. The addition of seeds with small size particles (fabricated by conventional solid-state method) and large size particles (molten-salt method) to the matrix was performed. The high relaxor phase ST26 matrix is combined with relaxor ferroelectric (RF) ST10 seed to fabricate composite ceramics, where large (r-ST26-ST10) and small (t-ST26-ST10) grain ceramics exhibited frequency-related dielectric properties and a large strain response at a low triggering electric field. The effects of the seed contribution with different soaking times on the crystal structure, dielectric permittivity, polarization, and electric field-induced strain response of composites with the change in frequency were studied, and the dynamics of the polarization kinetics were analyzed.

## Results and Discussion

**Figure 1:**
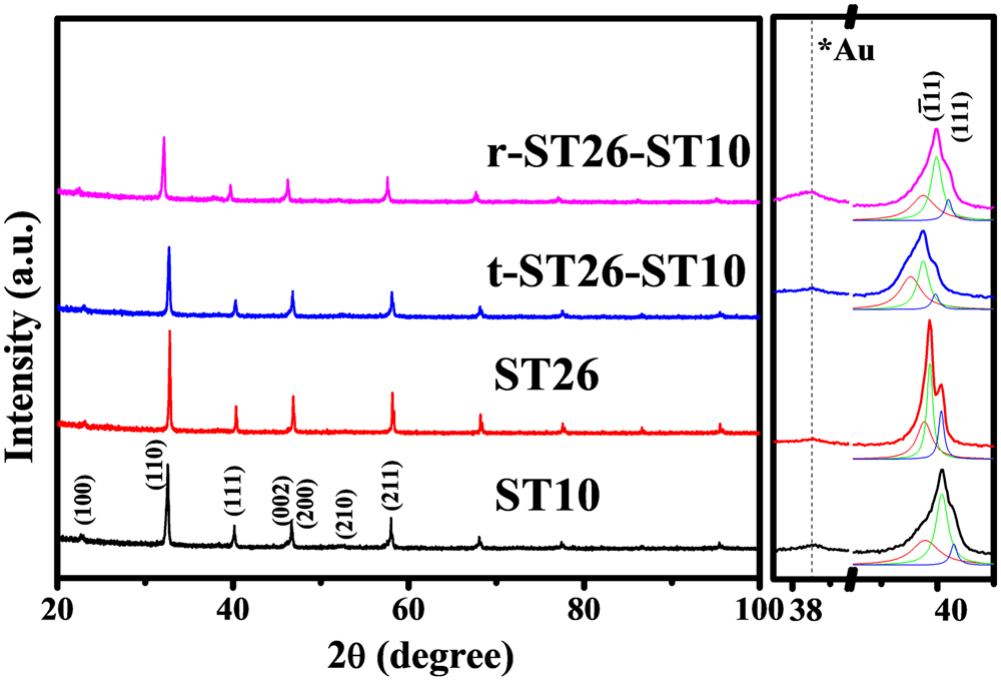
X-ray diffraction patterns of the seed ST10, matrix ST26, t-ST26-ST10 and t-ST26-ST10 composites sintered for 4 h. (**a**) Detailed analysis was performed in the range of 39~41° corresponding to the peaks (111) to highlight the coexistence of the mixed pseudocubic and rhombohedral phases.

The X-ray diffraction (XRD) patterns of the sintered matrix, seed and composite sintered for 4 h are shown in Fig. 1. The XRD of the 4 h sintered specimen revealed a unique perovskite configuration with no impurity or secondary peaks. According to the XRD pattern, the ST concentration with a cubic phase in BNT (rhombohedral, Pm3m) can be used for indexing the hkl values (Panalytical-X’pert high score program). In the case of a composites, there is a change in the phase from ferroelectric rhombohedral to mixed rhombohedral and pseudocubic phase with the addition of a seed in the matrix. To further evaluate the coexistence of the relaxor (pseudocubic and rhombohedral) and ferroelectric (rhombohedral) phases, a detailed analysis was performed in the range of 39~41° corresponding to the peaks (111). The separation of peaks was observed with a 10% seed content. The broadening of the (111) peak in Fig. 1 of the composite shows the combination of both phases. The peaks in the XRD pattern shifted to lower angles with the composition, indicating lattice parameter and cell volume expansion. The changes in the lattice parameter and cell volume are attributed to the addition of Sr^2+^ ions with a larger ionic radius (1.26 Å) than bismuth (Bi^3+^) and sodium (Na^+^) (1.17 Å, 1.18 Å resp.). These changes in the lattice parameter and lattice energy favor a phase change to alleviate the perovskite structure^6,7^.

**Figure 2:**
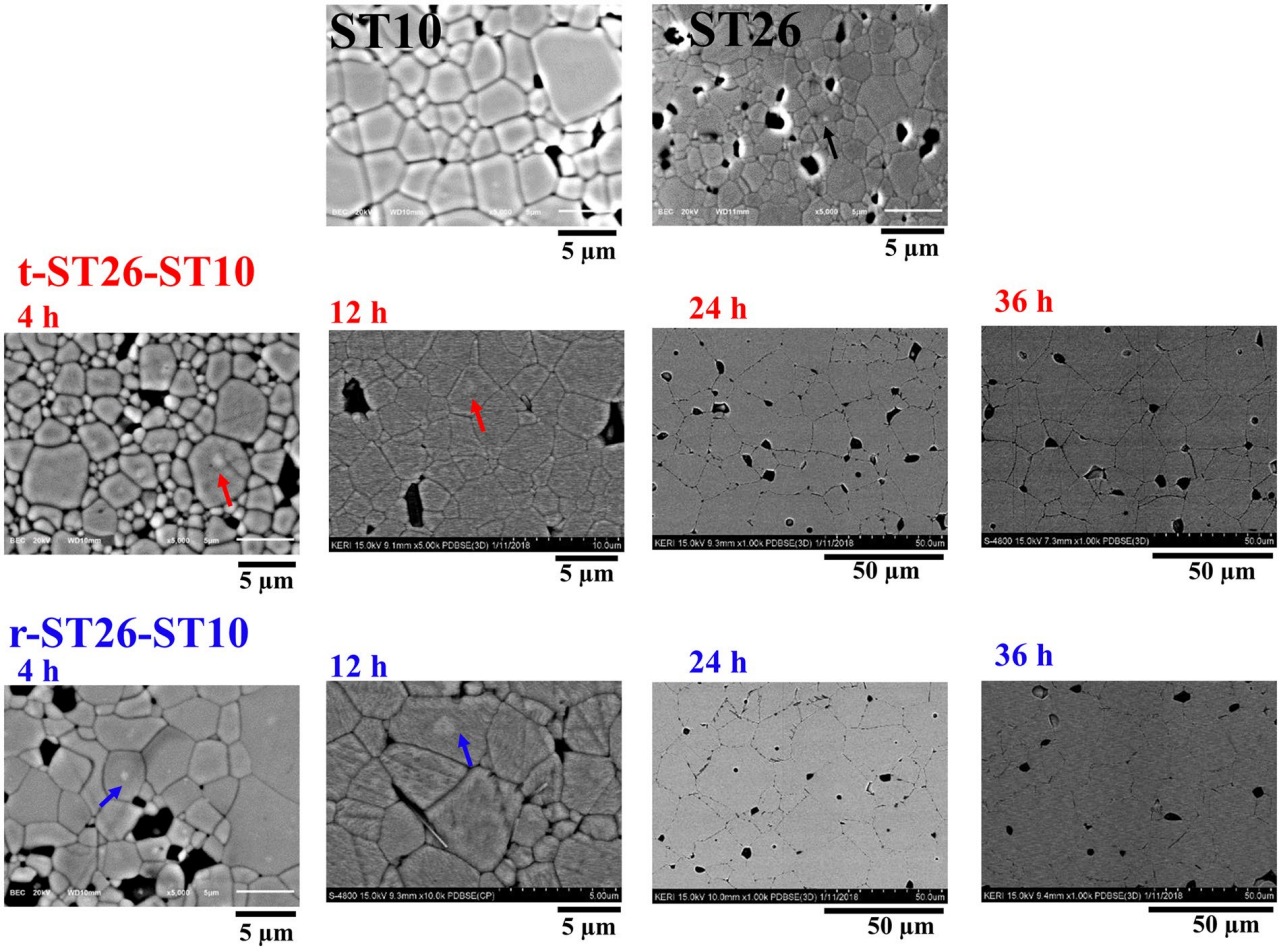
Backscattered microstructures of the polished surface of the seed ST10, matrix ST26, t-ST26-ST10 and t-ST26-ST10 composites sintered for different soaking times.

**Figure 3:**
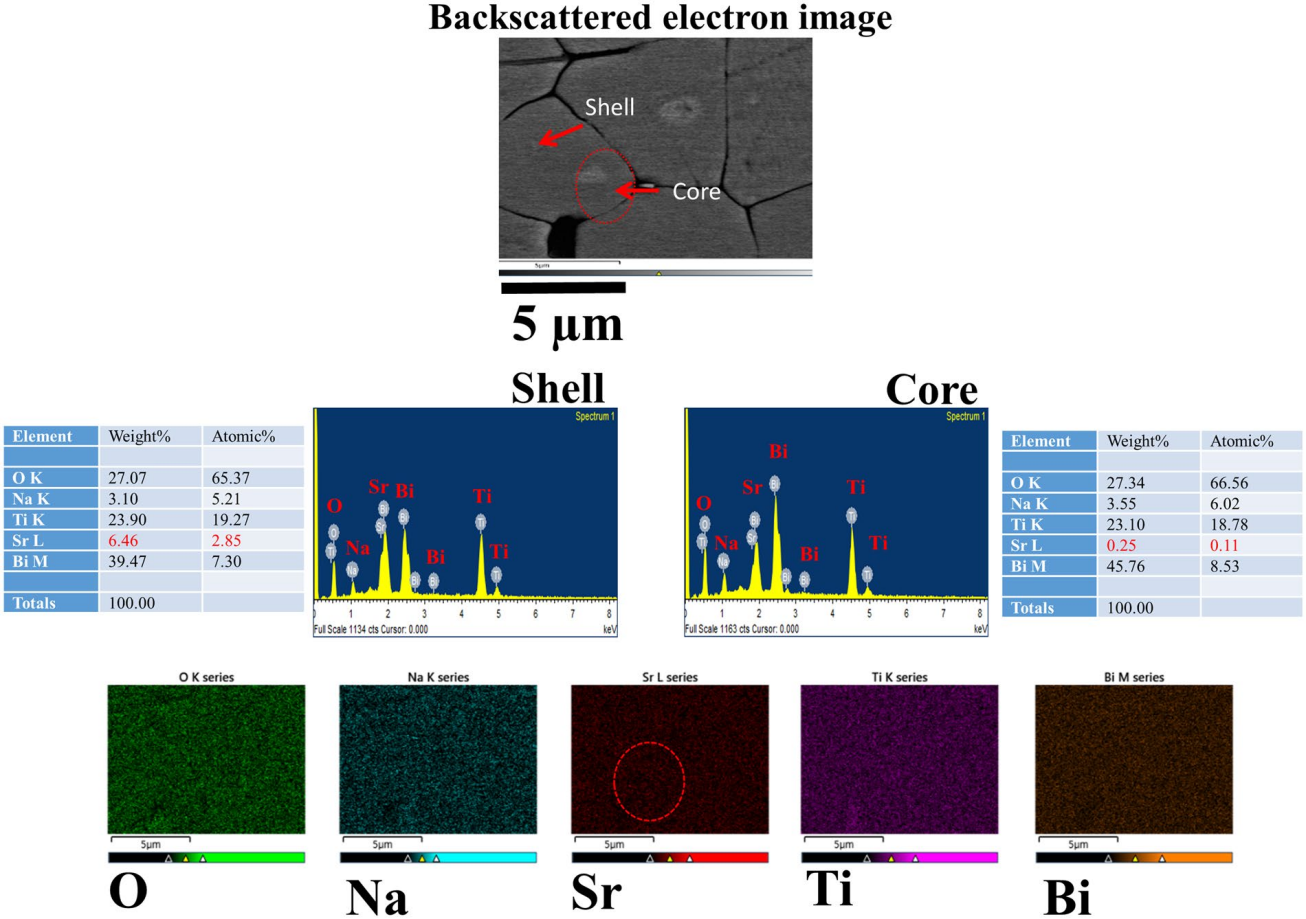
EDX analysis and compositional mapping of Bi, Na, K, Sr and Ti elements in the backscattered image of core-shell grain of r-ST26-ST10 composite sintered at 1150 °C.

**Figure 4:**
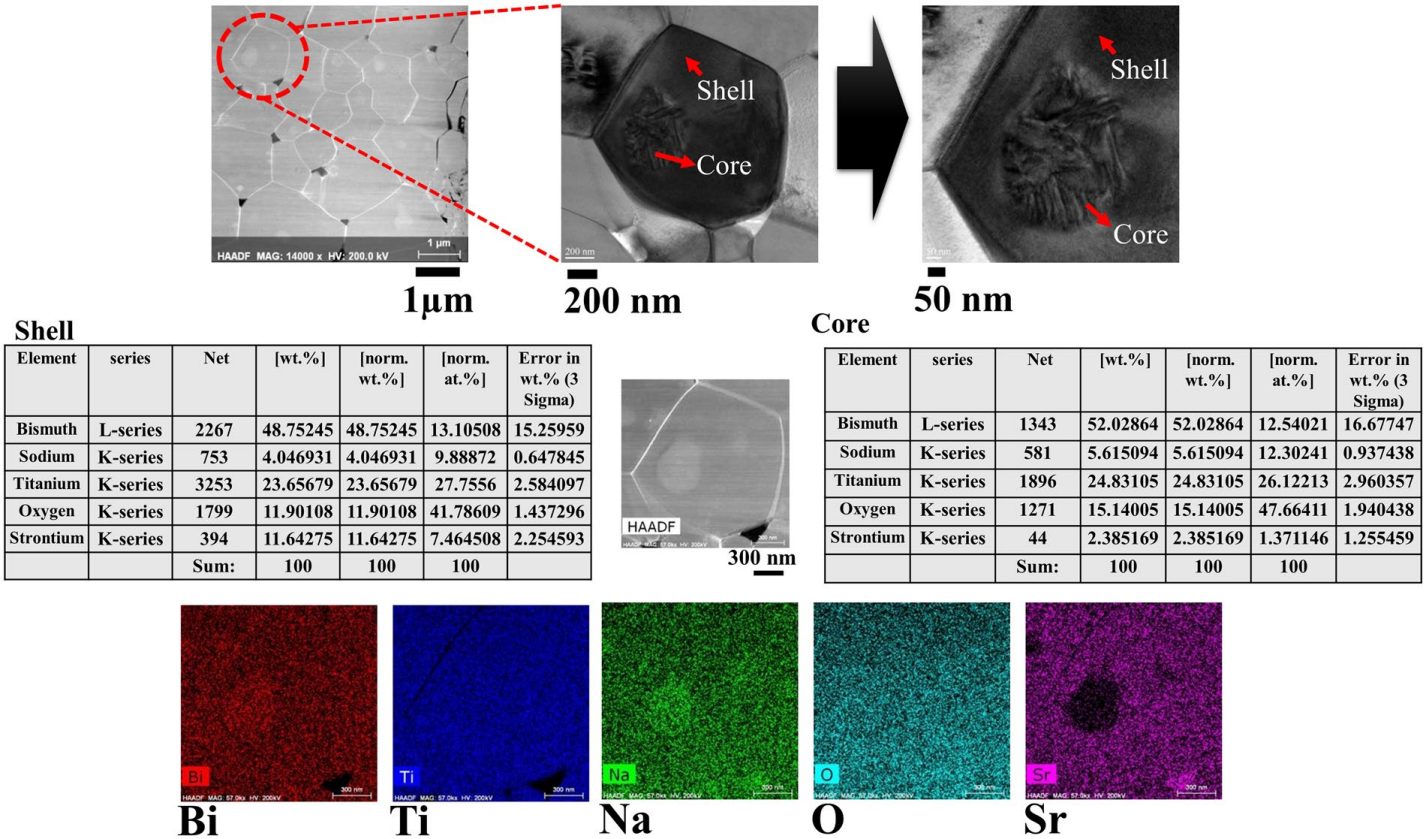
TEM images and compositional mapping of Bi, Na, K, Sr and Ti elements of the core-shell grain of t-ST26-ST10 composite sintered at 1150 °C.

The morphological characterization was performed by field emission scanning electron microscope (FE-SEM) and transmission electron microscope (TEM) for the composite samples. Figure 2 shows the microstructures of the polished surface of the matrix, seed, and composites obtained by FE-SEM in backscattered electron (BSE) mode sintered at 1150 °C with different soaking times. All samples were well sintered with a theoretical density of more than 95%. An increase in the grain size and a vanishing core shell structure were observed in the composites as a function of the increasing sintering time. In Fig. 2, there is no contrast in the ST10 seeds measured in the back-scattered electron image. In the case of a ST26 matrix, the difference in composition is visible due to the combination or existence of two phases and the core-shell structure. Therefore, the combination of different structures in a composite is clearly visible. The disappearance of the core revealed the homogenization of the thermally activated diffusion process of the seed and matrix with an increase in the sintering time from 4–36 h. In addition, the dissolution of the core resulted in the homogenization of the thermally activated diffusion process of the seed and matrix. Energy dispersive spectroscopy (EDX) was used to characterize the compositional difference. In Fig. 3, the images of the r-ST26-ST10 composite were further examined by EDX and mapping, which are performed for the analysis of the chemical composition. To further confirm the existence of the core-shell structure, TEM images were obtained for the t-ST26-ST10 composite, as shown in Fig. 4. EDX mapping revealed the origin of the core-shell structure, which has a BNT-rich core and ST-rich shell. Quantitative analyses illustrated that Sr and BNT are rich in both shell and core sections. And Bi content is higher than that of Sr in the core region which results in a brighter contrast of the core area as shown in Figs 2–4. In both composites with different grain sizes, the core did not vary in size, but the shell size dramatically changed with an increase in the soaking time.

**Figure 5:**
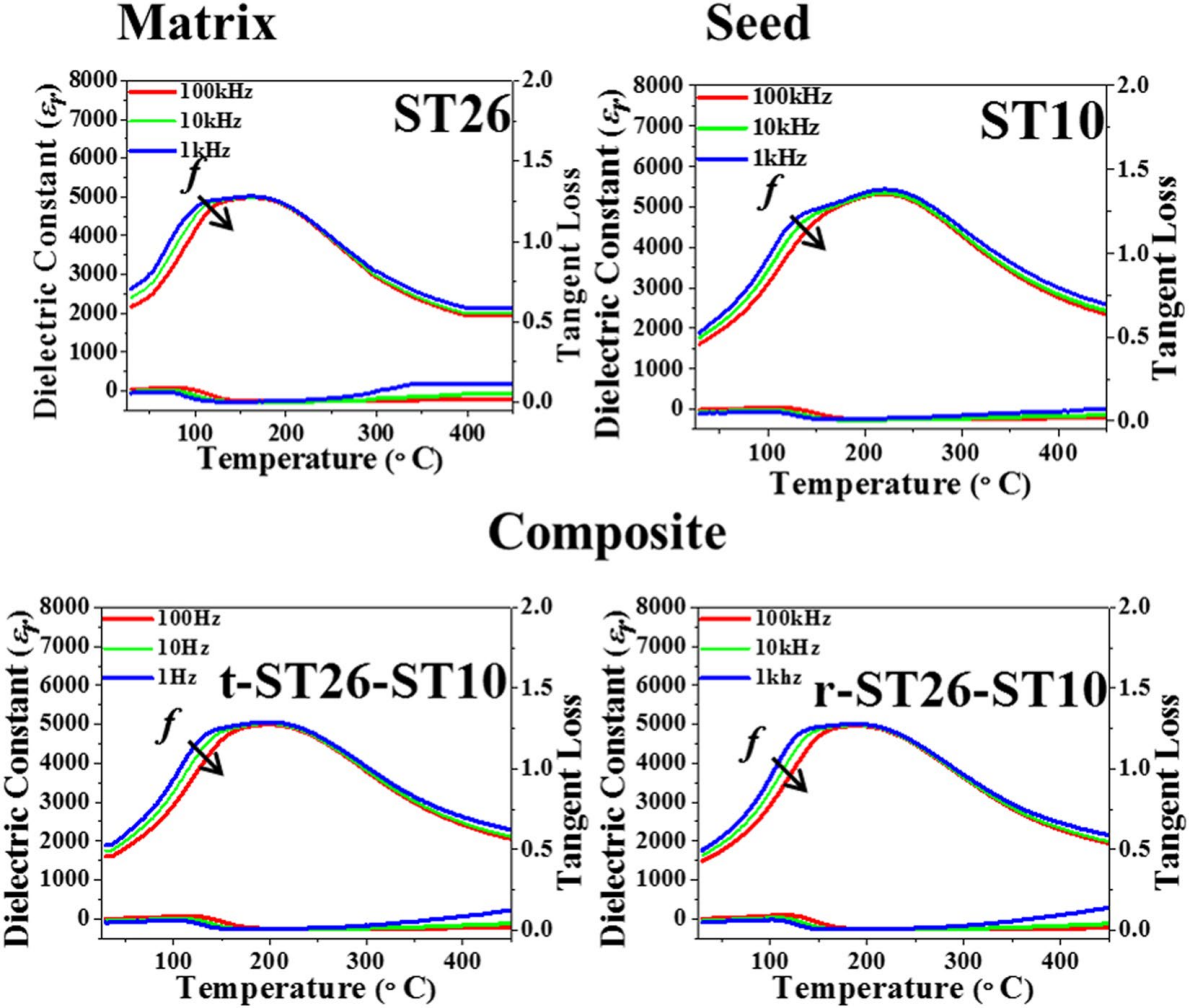
Temperature dependence of dielectric constant and dielectric loss of the matrix ST26, seed ST10, t-ST26-ST10 and t-ST26-ST10 composites for different frequencies.

Figure 5 displays the dielectric permittivity (Ɛ_r_) versus the temperature of the seed, matrix, and composite at various frequencies. The graph steadily widened with the increasing ST content along with the alteration of the peak on the low-temperature side. The high diffuseness in the dielectric permittivity indicates that the material in a high-temperature range (150–400 °C) has a higher degree of relaxation^21–23^. The related outcomes can be perceived as Ɛ_r_ behavior in the high-temperature range. In addition, the depolarization (T_d_) or transition temperature (T_F-R_) was not clearly identified for the ST content, especially in ST26^23,24^, which might be observed as a mixed state of FE and RF phases.

Table 1 shows the piezoelectric, electromechanical and dielectric properties of the seed, matrix, and composite. The piezoelectric constant (d_33_) decreased from 142 pC/N (ST10) to 8 pC/N (ST26). The electromechanical coupling factor (*K*_*p*_) value declined from 27.66% (ST10) to 14.96% and 15.01% (t-ST26-ST10 and r-ST26-ST10, respectively). The *K*_*p*_ value of ST26 cannot be measured due to the very small piezoelectric constant. In the case of the composites, the increasing behavior of the d_33_ value from 18–23 pC/N is due to the presence of the ferroelectric seeds sintered for 4 h.

**Table 1:** Room temperature dielectric constant (Ɛ_r_), Tangent loss, polarization (P), strain (S), piezoelectric constant (d_33_), normalized strain (d_33_*) and electromechanical coupling factor (*K*_*p*_) values of ceramic samples.

BNT-ST	Density (g/cm^3^)	Dielectric Constant (E_r_) at RT	Tangent Loss	P_r_ (µC/cm^2^)	P_max_ (µC/cm^2^)	S_max_ (%) (Bipolar)	d^*^_33_ (ρm/V)	d_33_ (pC/N)	K_p_ (%)
ST10	5.58	2169	0.05	30.5	39.4	0.09	175	142	27.66
ST26	5.62	2620	0.05	7.1	36.9	0.20	500	8	—
t-ST26-ST10	5.86	1760	0.06	9.7	32.2	0.30	890	23	14.96
r-ST26-ST10	5.85	1852	0.04	7.5	38.9	0.26	700	18	15.01

**Figure 6:**
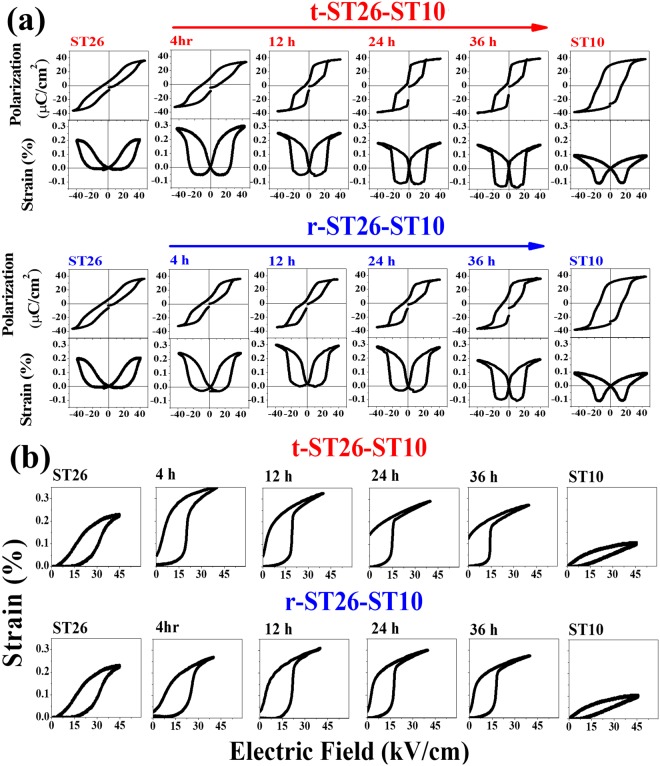
(**a**) Electric field induced polarization hysteresis loops, Bipolar and (**b**) unipolar strain of the matrix ST26, seed ST10, t-ST26-ST10 and t-ST26-ST10 composites sintered for different soaking times measured at 0.1 Hz.

**Figure 7:**
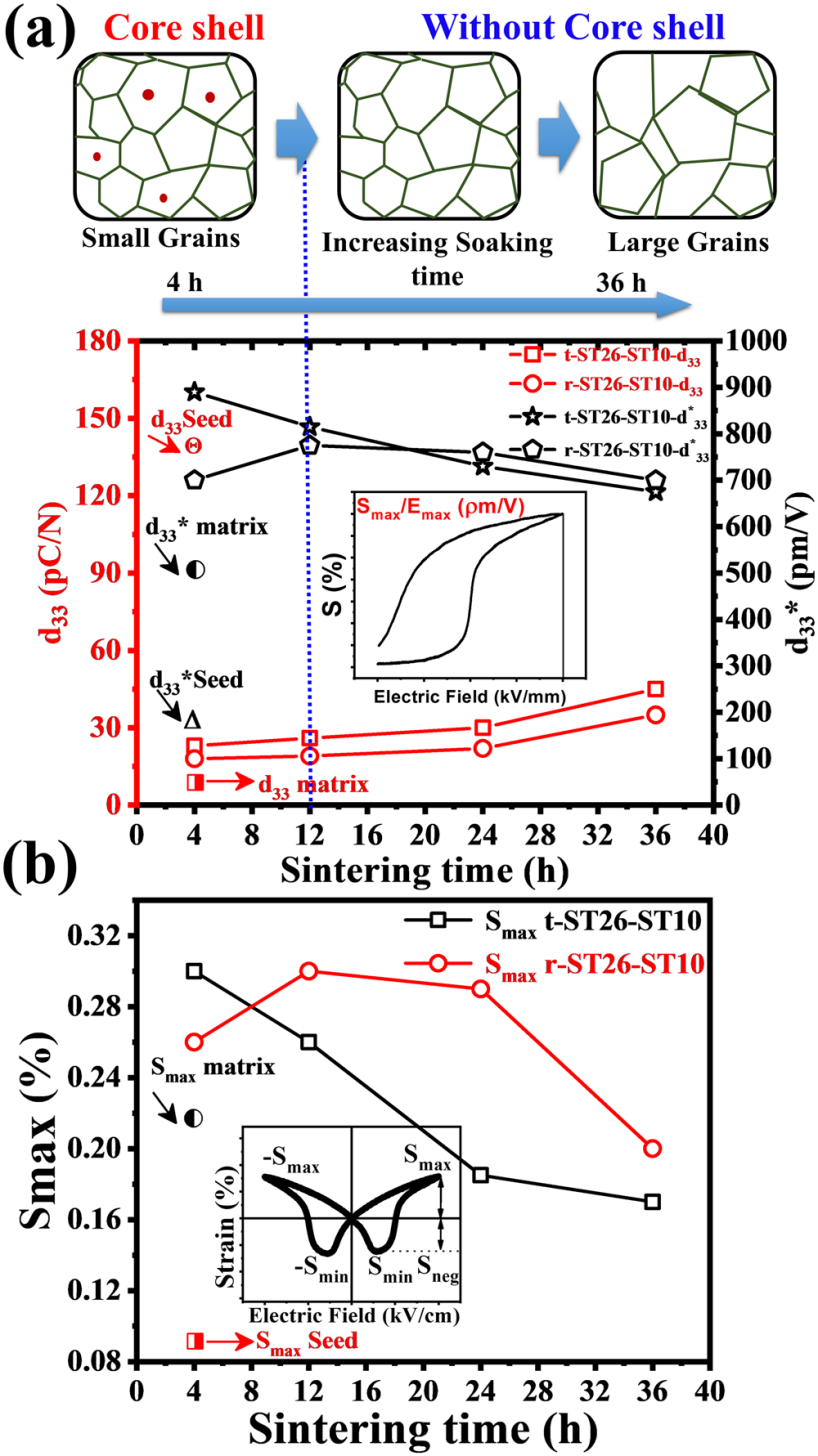
(**a**,**b**) Piezoelectric constant d_33_, normalized strain d_33_*, and maximum bipolar strain (S_max_) as a function of different soaking times of the composites. Contribution of the core of a ferroelectric seed in a matrix is analyzed for the evolution of polarization, strain and phase transition.

The polarization-electric field (P-E) curves of the seed, matrix, and composite were measured at 0.1 Hz, as shown in Fig. 6(a,b). Room-temperature strain (bipolar and unipolar curves) for the ST26 composite with the seed ST10 were obtained for different soaking times. The P-E loop of the t-ST26-ST10 composite shows a pinched-type curve with a decrease in the remnant polarization (P_r_ from 9.7 to 8.7 µC/cm^2^) and coercive field (E_c_ from 1 to 0.4 kV/cm) due to the change from a ferroelectric to relaxor nature. The opposite trends were also observed and were more prominent in the r-ST26-ST10 composite (P_r_ from 7.3 to 14.4 µC/cm^2^ and E_c_ from 0.4 to 1 kV/cm). However, the saturation polarizations were almost the same as the soaking time increased for both types of seed content. In the case of bipolar strain, maximum strain (S_max_) of 0.30% and 0.26% were achieved at 40 kV/cm for t-ST26-ST10 and r-ST26-ST10 composites sintered for 4 h. Overall, the field-induced strain tends to decrease with increasing the soaking time. The increase in the soaking time accelerates the phase transformation from short-range ordering RF phase to long-range ordering FE phase or vice versa. Under a unipolar strain, the minimum triggering field required to achieve the high strain is 25–30 kV/cm. A large dynamic normalized strain (d_33_*) is achieved in case of t-ST26-ST10 with ~890 pm/V and r-ST26-ST10 with ~700 pm/V at 40 kV/cm with soaking time of 4 h. The highest unipolar d_33_* is attained in t-ST26-ST10 (~890 pm/V) at soaking time of 4 h, while in r-ST26-ST10 (~775 pm/V) at soaking time of 12 h. With the further increasing soaking time, the negative strain also increased due to the absorption of the seed into the matrix, resulting in a homogenous solution and the disappearance of the core-shell structure, as shown in Fig. 2. The d_33_, d_33_*, and S_max_ values of the seed, matrix, and composite are shown in Fig. 7(a,b). In the case of the t-ST26-ST10 composite, the d_33_, d_33_*, and S_max_ values show a significant decrease with increasing the soaking time compared to those of the r-ST26-ST10 composites. This finding shows the considerable contribution of the core of a ferroelectric seed in a matrix to a significant enhancement in the polarization, strain and phase transition.

**Figure 8:**
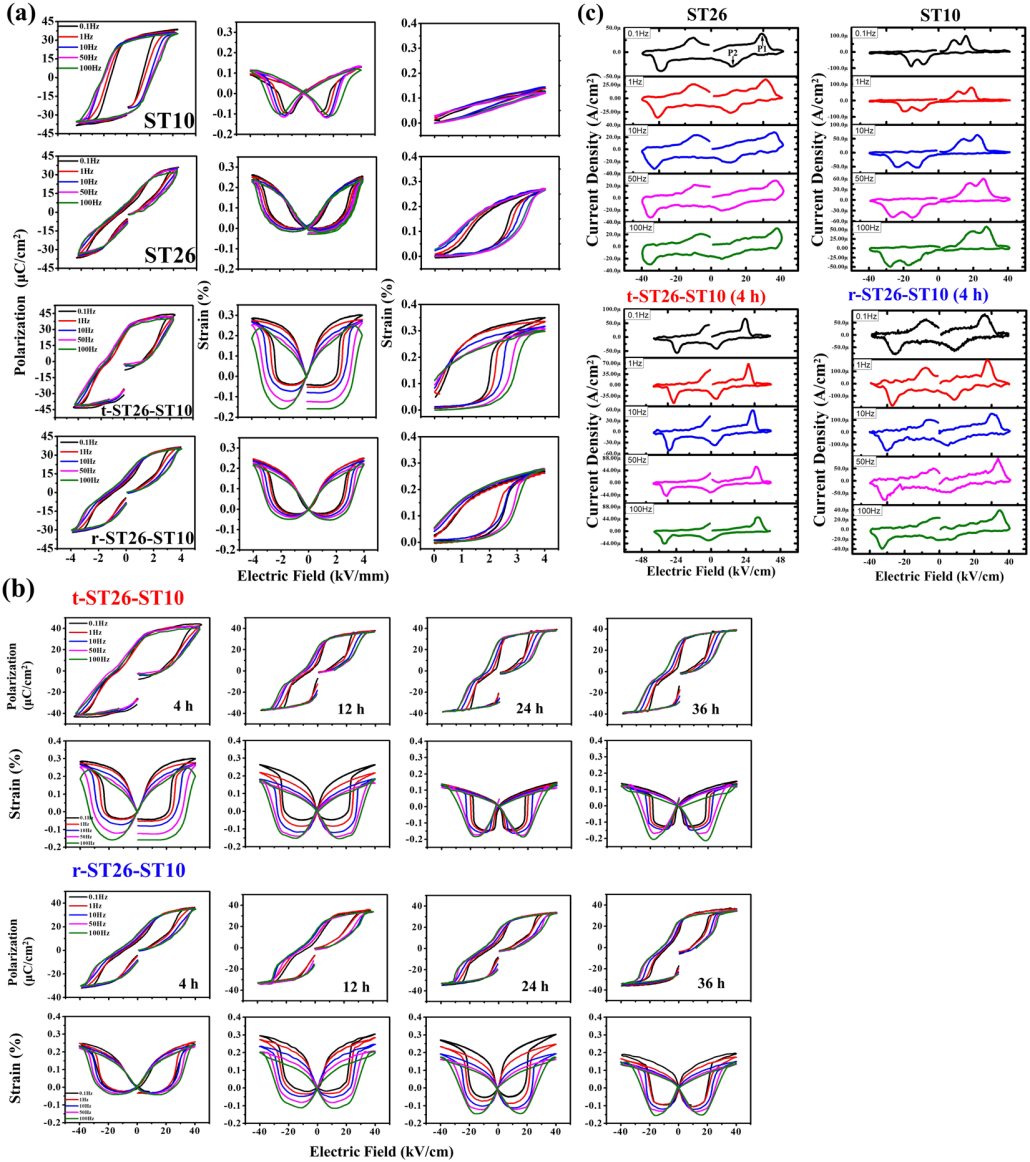
(**a**–**c**) Electric field induced polarization hysteresis loops, Bipolar and unipolar strain and corresponding current density curves of the sintered for different soaking times measured at different frequencies.

Figure 8(a–c) illustrates the polarization, strain and current density (J-E) curves with an increase in frequency in the range of 0.1 to 100 Hz. The long-range ordering ferroelectric ST10 (seed) shows a typical ferroelectric curve. With the rise in the ST addition in BNT (matrix), the change from ferroelectric to relaxor (pinched-type) curve is observed. In the case of the frequency, the degradation is not severe in the ferroelectric seed ST10 and relaxor ST26 matrix. However, in the composite of t-ST26-ST10, the frequency-dependent polarization has a higher degradation with large P_r_ and E_c_ values due to the small grain size with the increased soaking time, as shown in Fig. 8(b). Therefore, the large grain size composite of r-ST26-ST10 shows a smaller degradation of the polarization and strain with frequency. To further examine the field-induced phase transition, the J-E curves were obtained to determine the behavior of the seeds in ST26, as shown in Fig. 8(c). There are four peaks detected on most of the curves, demonstrating the existence of the relaxor (RE) to ferroelectric (FE) phase transition with a reverse transition upon the application and removal of an applied field. In this case, sharp current peaks (denoted as P1 and P2) describe the domain switching observed when the applied voltage reaches E_c_. In ST10, the relaxor characteristic leads to irreversible behavior in the ferroelectric phase when the applied field approaches the E_c_. Therefore, two sharp current pulses are visible in the J-E curve due to the kinetic behavior of the random field’s effect^4,25,26^. The appearance of two broad pulses is related to the long-range ordering ferroelectric phase of seed and indicates the coexistence of non-ergodic and ergodic relaxor phases. These pulses move with the ST content and are associated with the disruption of the lattice parameter and long-range ferroelectric behavior in lower polarization states^25–29^.

The high, characteristic ferroelectric small grain size of ST10 with an increase in the soaking time shows a higher degradation of strain with frequency, as shown in Fig. 8(b). With an increase in the soaking time, the composite shows a higher negative strain and degradation with frequency. These results show that the polar ferroelectric phases with macro-size domain structures are generated, leading to the emergence of the second polarization broad current peak in the J-E curve.

### Polarization kinetics

The kinetics of the composites are mainly associated with the nucleation of the ferroelectric seed and its growth in the relaxor matrix. The existing ferroelectric phase has a high polar characteristic that leads to the most prominent nucleation site for FE in the RE matrix during the phase transition upon the application of an electric field. The nucleation and growth of FE domains are likely to occur in the matrix near the FE grains. Therefore, ferroelectric grains exhibit high degradation in the polarization and strain with frequency. There is the possibility of domain orientation upon removal of the electric field, and the internal field generated by the ferroelectric seed has an important role in the degradation of the polarization and strain. A matrix with a high relaxor characteristic returns to its original position after the removal of ‘E’. In the case of the seed, the ferroelectric behavior causes the domains to partially return to the original or virgin state after the removal of the applied voltage, ‘E’. This ferroelectric seed hinders the movement of the domain wall from long-range ordering ferroelectric behavior to short-range ordering during the reverse transition process. Thus, severe degradation with frequency occurs for bipolar and unipolar strain of the ferroelectric seed.

This possibility was further evaluated with the KAI model and the switching time to understand the kinetics of the seed in the phase transformation of the composites. The reversible switching of the seed, matrix, and composites provided enough information for the kinetics of the composites to be measured by the PUND test.

**Figure 9:**
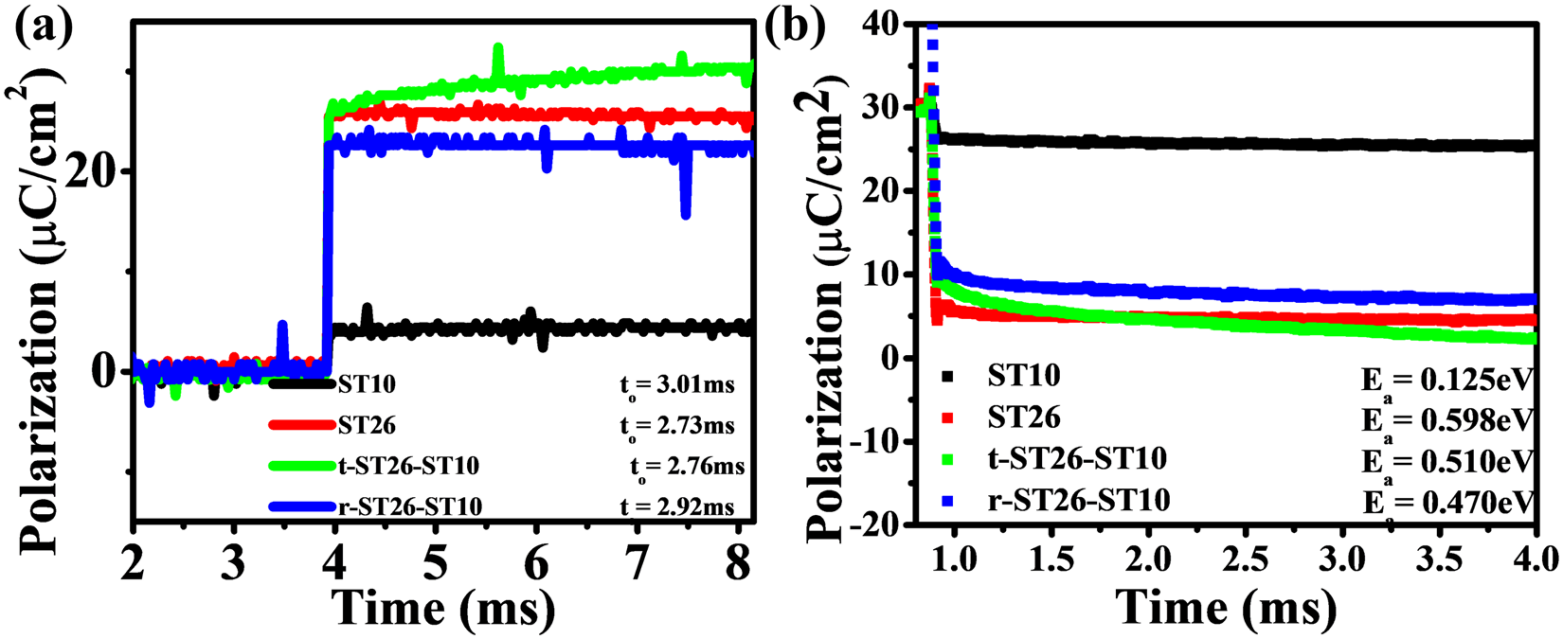
(**a**,**b**) Switching polarization (P) versus characteristic switching time (t_o_) and activation energy (E_a_) at 1 Hz for the seed ST10, matrix ST26, t-ST26-ST10 and t-ST26-ST10 composites.

PUND (Positive up Negative Down) measurements on the seed, matrix, and composite were performed. The results are shown in Fig. 9(a,b). In this measurement, the polarization was completed within 4 ms to 8 ms. The lines were fitted using the KAI (Kolmogorov-Avrami-Ishibashi) model^29,30^. Figure 9 is the fitting, which shows that the measured data were defined by the KAI model.

1 $${P}_{sw}(t)=2{P}_{r}[1-exp(-{(t/{t}_{o})}^{n})]$$

P_sw_ = switching polarization, P_r_ = remnant polarization, t_o_ = characteristic time, t = switching time, n = geometric dimension for domain growth.

Further analysis of ‘t’ can be achieved with a graph of *t*_*o*_ versus *1/E*, as shown in Fig. 8. The linear relationship of Mertz’s law shows the characteristic switching time^31^,

2 $${t}_{o}=t\,exp(-{E}_{a}/RT)$$

However, t_o_ = characteristic time, *E*_*a*_ = activation energy, R = Boltzmann constant, T = temperature.

All specimens showed polarization with the saturation behavior versus time for the applied driving field of 4 kV at 1 Hz. Figure 9 shows the switching time of the ceramic in the reversal of the electric field, ‘E’. By applying the ‘E’, the domains are oriented in the direction of E. However, upon the removal of E, the ferroelectric domain is not completely reversed to its original or virgin position. Some of the domains are reversed, and others are only partially reversed. In the case of ST26 (2.73 ms), the switching time decreased due to the complete reversibility of the domain to its original state compared to that of ST10 (3.01 ms). The switching time characteristic of r-ST26-ST10 (t_o_ 2.92 ms) is higher than that of t-ST26-ST10 (t_o_ 2.76 ms). The existence of a larger ferroelectric phase shifted the switching time to a higher value. The characteristic relaxation time was obtained by fitting the results of Fig. 9 and eq. (1) (KAI model). Fitting parameter ‘n’ used to fit eq. 1 and has a fixed value of 3^29–33^. The fitted data derived from eq. (2) were plotted with the measured data.

**Figure 10:**
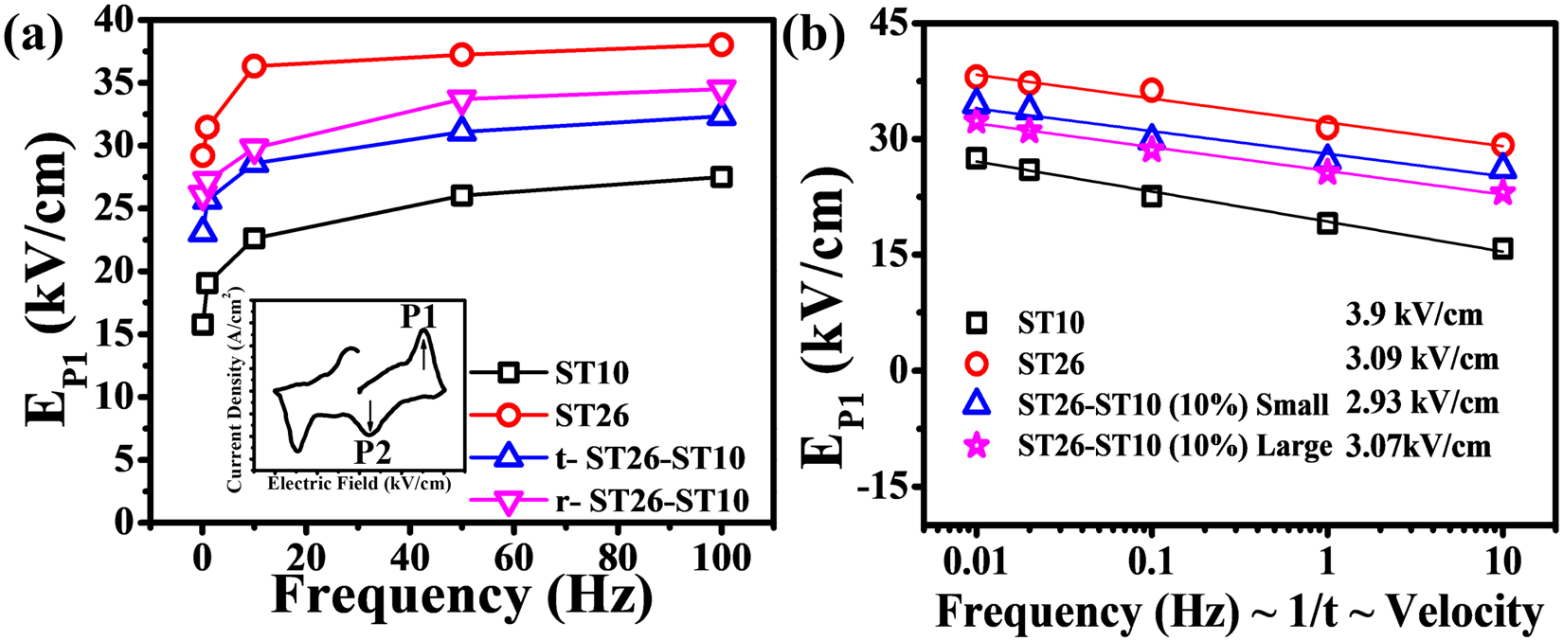
(**a**,**b**) Electric field (E_P1_) and domain wall velocity (v) versus frequency (Hz) for the seed ST10, matrix ST26, t-ST26-ST10 and t-ST26-ST10 composites.

The activation energy (E_a_) of the seed, matrix, and composite were obtained from the linear relation of Mertz’s law. The activation energy was 0.125 eV for ST10 and 0.598 eV for ST26, as shown in Fig. 9(a,b). These E_a_ values suggest that the ST addition lowered the thermal energy obstacle for the conversion from ferroelectric to relaxor phase. An increase in the activation energy suggests a decrease in the relaxation time. This will cause the domain wall movement to increase with the frequency. Therefore, the relationship between the frequency and electric field related to the phase change can be examined, as shown in Fig. 10(a). At a high frequency, there is less time required for the domain to transform to its original position. The average domain wall velocity (v) was measured with the modified equation of Mertz’s law at different frequencies. The domain wall velocity of the seed, matrix, and composite was measured by a modified equation of Mertz’s law. The equation for the domain wall velocity^30^ is

3 $${\rm{Velocity}}\,{\rm{v}} \sim 1/{{\rm{t}}}_{{\rm{o}}=}1/{t}_{\infty }\exp (-\,{\rm{\delta }}/{{\rm{E}}}_{1})$$

where

$$\begin{array}{c}\,{\boldsymbol{\delta }}={\rm{activation}}\,{\rm{field}}\\ {{\bf{E}}}_{{\bf{1}}}={\rm{electric}}\,{\rm{field}}\end{array}$$

Further exploration of the change in the electric field with the frequency in the J-E curve gives the domain wall velocity (v). The linear relation of Mertz law is presented in eq. 3. Figure 10(b) displays the decreasing trend for the domain wall velocity with the frequency.

These outcomes indicate that the ‘v’ of ST10 is higher than that of matrix ceramics. It has already been mentioned that ST10 has a high ‘*E*_*a*_’ and low ‘t_o_’. Thus, the slow movement of a domain wall in ST10 is related to the characteristics of the ferroelectric phase. These analyses show that the frequency-related properties are highly affected by the presence of the seed, leading to the degradation of the piezoelectric and ferroelectric characteristics. From ST10 to ST26, the domain wall velocity increases with the addition of ST content. Additionally, the E_a_ and t_o_ values reveal the seed corresponds to the decrease in the domain walls velocity. In the case of a composite, the ferroelectric seed is responsible for the degradation of the polarization and strain. The small grain size of the seed leads to a higher degradation of the polarization and strain compared to that with a large grain size. The movement of the domain wall velocity is associated with the presence of seeds with a large grain size, giving rise to less degradation of the frequency dependence polarization and strain. This occurs because the internal random fields created by the seed, slow down the effect of a reversion of induced long-range ferroelectric ordering to short-range ordering of relaxor phase with the frequency.

The frequency-related electromechanical properties of r-ST26-ST10 and t-ST26-ST10 composites at different soaking times were investigated. ST26 composite ceramics with different sizes of the ferroelectric seed ST10 were successfully synthesized by a conventional solid-state reaction and molten-salt method. The polarization and electric field-induced strain increased with only 10% addition of seeds to the matrix and overall decreased with the increasing soaking time for t-ST26-ST10 and r-ST26-ST10. The degradation of the polarization and electric field-induced strain with the frequency was investigated in detail. The critical composition, ST26-ST10 with 10% seed in the composite, resulted in a high P_r_, S_max_, and d_33_* values with an enhanced phase transition. From the study of the polarization and strain with the frequency, a ferroelectric addition prompted the stability corresponding to the coexistence of FE (seed) and RE (matrix) phases in a composite. The electromechanical properties of the composites increased until the optimum composition and soaking time for the 10% large grain size seed content was reached, and then, they gradually decreased and changed to the FE phase with the increased soaking time of the composite, driving the absorption of the two phases in the ABO_3_ perovskite structure. The decreasing trend of the activation energy calculated using the KAI relationship suggests that a faster domain velocity was generated by increasing the seed content in a ST26 matrix. This result suggests that the ST26 composite is a promising candidate for Pb-free electromechanical applications.

## Material and Methods

### Sample Preparation

These ceramics were created by a conventional oxide route using reagent grade oxides and carbonates (Alfa Aesar GmbH, Karlsruhe, Germany). Bi_2_O_3_ (99.975%), Na_2_CO_3_ (99.9%), TiO_2_ (99.9%), and SrCO_3_ (99%) were mixed according to the (1-x) Bi_0.5_Na_0.5_TiO_3_–xSrTiO_3_ (x = 0.10–0.26) stoichiometric formula.

Different seed sizes were prepared by a solid-state reaction (small size) and molten-salt method (large size). The solution was ball milled for 24 h in ethanol. Afterward, powders calcined at 850 °C for 2 h, ball milled with ethanol and dried at 90 °C. The matrix and seeds were selected based on the compositions 0.74 Bi_0.5_Na_0.5_TiO_3_–0.26SrTiO_3_ and 0.90 Bi_0.5_Na_0.5_TiO_3_–0.10SrTiO_3_. The components were mixed together according to the volumetric ratio (9:1). The dried powder was mixed with a PVA binder to create pellets. The powder was pressed into green disks with a diameter of 12 mm and a thickness of 2 mm. The pellets were calcined at 550 °C for 2 h and then sintered at 1150 °C for several sintering times ranging from 4–36 h. To reduce the evaporation of Bi and Na elements (volatile), the green disks were concealed with powder possessing the same content. The sintered pellets were refined and used to create an electrode with silver paste. Then electrodes were heated at 700 °C for 30 min.

### X-ray diffraction

The X-ray diffraction (XRD) patterns were measured in a Panalytical X’pert PRO MPD system equipped with an X’celerator detector and a graphite monochromatic (Cu Kα1 radiation, 1.54056 Å). The diffraction patterns were recorded from 20° to 100° (2θ) with an angular step interval of 0.01.

### Scanning and Transmission Electron Microscopy

FE-SEM (Hitachi FE-SEM S4800, Japan) was used for the morphological characterization. The FE-SEM has an energy-selective backscattered (EsB) detector capable of detecting the smallest differences in material composition. Density measurements were achieved based on the Archimedes principle. For the TEM studies, a specimen was prepared by the conventional method. TEM (JEM–3000F, JEOL, Tokyo, Japan) with selected area electron diffraction (SAED) was performed using a transmission electron microscope equipped with a field–emission gun. TEM samples were prepared by the conventional method, including polishing, dimpling and argon ion-milling. The sample was annealed at 250 °C to reduce the residual stress that accumulated during the polishing process.

### Electrical Analysis

A Precision LCR Meter 4192A (Hewlett Packard Corporation, Palo Alto, CA) was used for the measurements of the dielectric constant with the temperature. The electrical coupling factor was measured using an IEEE standard. P-E hysteresis loops were measured using a modified Saw-Tawyer circuit at 0.1–100 Hz. The PUND (positive up negative down) test was measured using a modified Saw-Tawyer circuit at 4 kV at a different frequency. Field-induced strains were measured using a contact-type displacement sensor.
